# Endoscopic Recanalization of a Long Segment Esophageal Obstruction Using the Combined Antegrade and Retrograde Rendezvous Procedure

**DOI:** 10.14309/crj.0000000000001754

**Published:** 2025-07-03

**Authors:** Himsikhar Khataniar, Katherine Albus, Inanc S. Sarici, Sven E. Eriksson, Shahin Ayazi

**Affiliations:** 1 Department of Medicine, Allegheny Health Network, Pittsburgh, PA; 2 Foregut Division, Surgical Institute, Allegheny Health Network, Pittsburgh, PA; 3 Chevalier Jackson Research Center, Western Pennsylvania Hospital, Allegheny Health Network, Pittsburgh, PA; 4 Department of Surgery, Drexel University, Philadelphia, PA

**Keywords:** esophageal stricture, endoscopic dilation, rendezvous technique, stricture management

## Abstract

The combined antegrade and retrograde endoscopic rendezvous technique effectively restores patency for esophageal short-segment obstructing strictures (<3 cm). However, long-segment strictures typically require complex surgery, with endoscopic management rarely reported. We report a 44-year-old man with a 9 cm esophageal obstruction due to peptic stricture who was at high risk of esophageal resection due to severe cardiac disease and prior abdominal surgeries. He underwent successful recanalization using a rendezvous technique. Serial dilations with subsequent esophageal stenting restored luminal patency. This case highlights the feasibility of an endoscopic approach as a safe and effective alternative to surgery in long-segment peptic strictures.

## INTRODUCTION

Management of esophageal strictures with complete luminal obstruction is a challenge. The combined antegrade-retrograde endoscopic rendezvous technique is effective in restoring patency for short-segment obstructed strictures (<3 cm).^1,2^ However, endoscopic management of long-segment strictures (>3 cm) is less well documented, often requiring complex surgery, such as esophagectomy.^3,4^ This case demonstrates the successful use of the rendezvous technique in a patient with a 9 cm completely obstructive esophageal stricture.

## CASE REPORT

**Figure 1.**
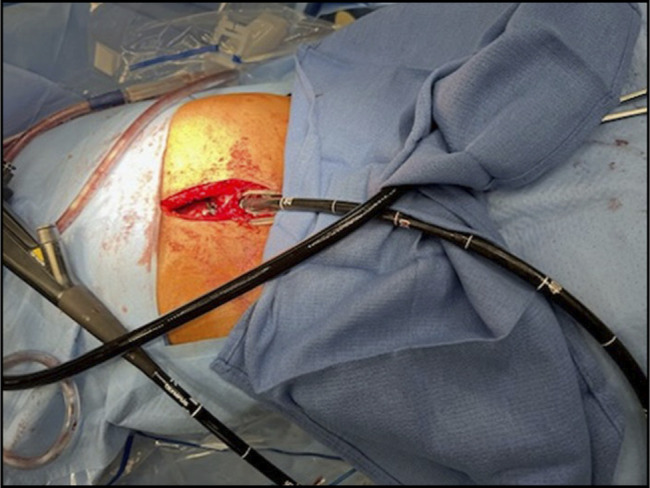
Retrograde endoscopic access was achieved through an upper midline abdominal incision.

A 44-year-old-man with seizure disorder, complex cardiac disease (near-total left anterior descending coronary artery stenosis on dual antiplatelet therapy), and history of abdominal surgeries for gastroschisis and intestinal malrotation was referred for complete esophageal obstruction, preventing oral intake or saliva management. Evaluation at another center revealed total luminal obstruction from long-segment stricture with failure to pass a guidewire. A jejunal feeding tube was placed, and, as he was not a candidate for esophagectomy, he was referred for endoscopy management. We planned a combined antegrade-retrograde endoscopic approach. Endoscopy confirmed complete obstruction at 30 cm. Abdominal access was obtained through an upper midline incision, followed by extensive adhesiolysis and gastrostomy for the retrograde approach (Figure 1).

**Figure 2.**
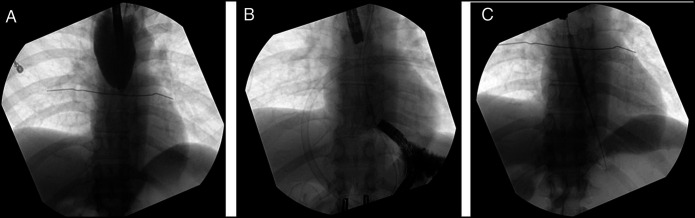
(A) Initial fluoroscopy demonstrated a narrowing at the distal esophagus, with no passage of contrast through the affected segment. (B) Fluoroscopic imaging confirmed the sagittal and coronal alignment of both endoscopes, demonstrating a 9 cm axial gap between their tips. A soft tip guidewire was successfully advanced distally across the stricture after multiple attempts. (C) Sequential antegrade and retrograde dilation was performed using 6–8 mm through-the-scope balloons under fluoroscopic guidance.

**Figure 3.**
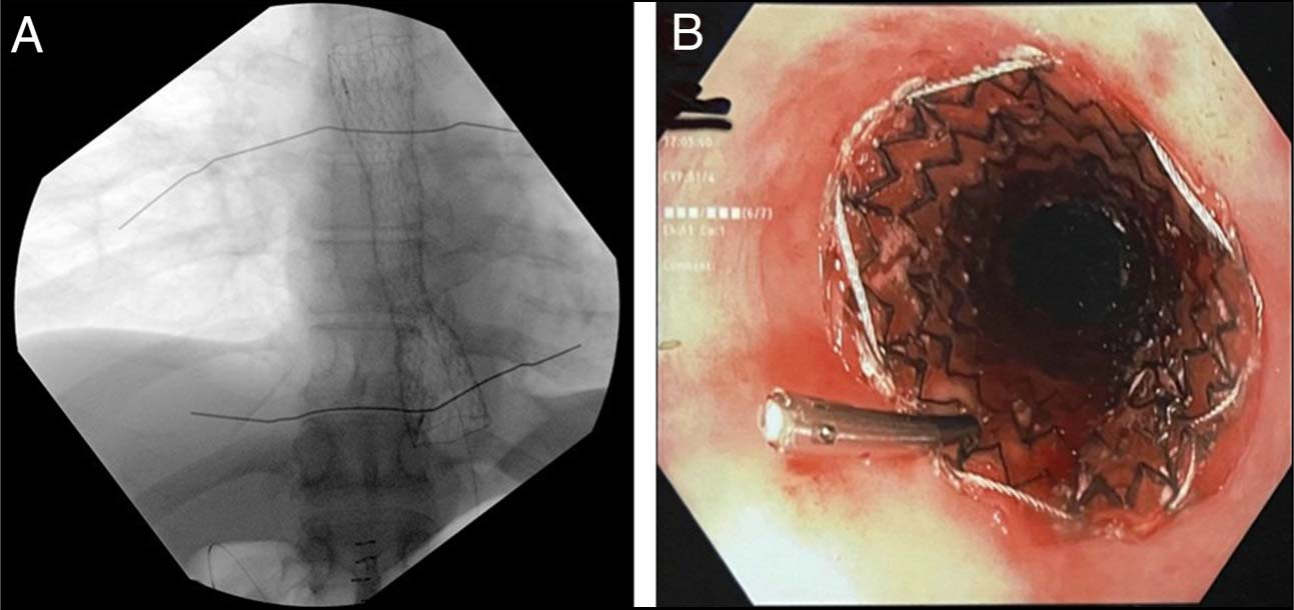
(A) Final fluoroscopic assessment confirmed the correct positioning of the esophageal stent. (B) Fully covered self-expandable metallic esophageal stent was deployed and secured with 2 Resolution Clips (Boston Scientific, Boston, MA).

Various guidewires, including angled and stiff wires, were initially attempted but were found to coil and unable to traverse the stricture. Fluoroscopic imaging demonstrated a narrowing at the distal esophagus, with no passage of contrast through the affected segment (Figure 2). Alignments of the endoscopes in both sagittal and coronal planes were then confirmed under fluoroscopic guidance, revealing a 9 cm axial distance between the endoscopic tips (Figure 2). Sharp and blunt probing was performed at both ends using soft-tip guidewire, pediatric biopsy forceps, and 19 G fine-needle aspiration (FNA) needle. Once the distal tip was visualized through the retrograde scope, the guidewire was grasped and pulled, establishing an intraluminal path (Figure 2). Antegrade and retrograde dilation was performed using 6–8 mm through-the-scope balloons under fluoroscopy (Figure 2). Contrast imaging confirmed passage into the stomach without perforation. An 8F nasogastric tube was placed to maintain patency. A 30F gastrostomy tube was placed through gastrostomy site to facilitate future intervention. Given the complexity of his care, the patient remained in hospital and 3 additional dilations were performed 4 days apart. Once 13 mm luminal patency was achieved, a fully covered 19 × 120 mm esophageal stent was placed and secured with 2 Resolution Clips (Boston Scientific, Boston, MA) to allow safe discharge and no concern for salivary secretion management or nutritional support (Figure 3). The stent was upsized 3 weeks later to 23 mm. The patient subsequently referred for management of left anterior descending coronary artery stenosis. During follow-up, the patient was monitored monthly with upper gastrointestinal endoscopy and underwent sequential balloon dilations over 6 months while the esophageal stent remained in place.

## DISCUSSION

Esophageal strictures from various etiologies, including peptic injury, radiation, caustic ingestion, and surgery, can lead to complete obstruction, impairing oral intake and saliva management, and increasing the risk of aspiration pneumonia, malnutrition, and dehydration.^5,6^ The combined antegrade-retrograde rendezvous technique, first performed by van Twisk et al in 1998, is a minimally invasive approach to restore luminal patency.^7^ Case series and meta-analyses have demonstrated a nearly 90% success rate in short-segment obstructive strictures.^3,8,9^ However, its use in long-segment cases has been limited due to fibrosis, loss of landmarks, and technical challenges.^10,11^ Therefore, surgical management is typically preferred for long-segment obstructing strictures. Given our patient's complex medical history and high surgical risk, we used the rendezvous technique, demonstrating viability as an alternative for long-segment esophageal strictures in high-risk patients.

Establishing a path through a completely obstructed esophagus is especially challenging in long-segment strictures. In our case, after aligning both endoscopes under fluoroscopic guidance, we used sharp and blunt probing techniques with a soft-tip guidewire, pediatric biopsy forceps, and 19 G FNA needle to safely navigate the obstruction. Recently, endoscopic submucosal tunneling from both the per-oral (POETRE) and gastric sides has been described as an effective rendezvous approach.^12,13^ POETRE was not feasible in this case due to extensive fibrosis and obliteration of the submucosal plane, which precluded the ability to create a safe submucosal tunnel. In addition, endoscopic ultrasound-guided techniques have emerged as valuable tools for esophageal recanalization in complex cases.^14^

Serial endoscopic dilations were required to maintain esophageal patency once it was achieved.^15^ In this case, we performed gentle dilation using a through-the-scope balloon, gradually expanding it to 6–8 mm. This approach provides direct visualization of the stricture and controlled application of radial force, enabling early detection of tears and prompt deflation to help prevent perforation.

The role of stent placement in refractory benign esophageal strictures remains controversial, with studies reporting a wide range of efficacy from 21% to 94%; however, a recent systematic review and meta-analysis of 18 studies involving 444 patients found that the pooled clinical success rate of stent placement was only 40.5%.^16^^,^^17^ However, in this case, stent placement allowed safe discharge while ensuring continued esophageal patency, facilitating secretion management and enteral nutrition, all of which were essential for planning a subsequent cardiac intervention. In our case, serial inpatient dilations were performed to achieve a luminal diameter of 13 mm, followed by the placement of a fully covered esophageal stent. The stent was upsized to 23 mm 3 weeks later, enabling the patient to successfully tolerate oral intake without the need for jejunal feeding. A recent meta-analysis have reported the estimated 58.4% dysphagia improvement and 43.5% percutaneous endoscopic gastrostomy-free nutrition after combined anterior and posterior rendezvous procedure for complete esophageal obstruction.^9^ The American Society for Gastrointestinal Endoscopy states that most patients can tolerate a normal diet once the luminal diameter reaches 13–15 mm, and the Committee of the British Society of Gastroenterology strongly recommends dilation to >15 mm for sustained symptomatic relief.^18,19^ Similarly, a retrospective study of 88 patients with benign anastomotic strictures after esophagectomy suggests that dilating the lumen beyond 16 mm can extend the dysphagia-free interval and decrease the frequency of repeated dilations.^20^

Perforation and pneumomediastinum are major complications in restoring patency to long-segment esophageal obstruction. A meta-analysis of 19 studies (251 rendezvous procedures) reported a pooled perforation rate of 8% and a pneumomediastinum rate of 10%.^9^ In our case, no early complications occurred. However, recurrence is common, with a meta-analysis estimating a 78.9% repeat dilation rate after rendezvous procedures for complete esophageal obstruction.^9^ Several studies have identified risk factors for recurrence. A single-center retrospective study of 87 patients with benign esophageal strictures found that a luminal diameter of ≤13 mm predicted recurrence within a year after dilation (odds ratio [OR] 1.71; 95% CI: 1.17–2.51).^21^ Similarly, a study of 63 patients with radiation-induced strictures found that severe stenosis (lumen <9 mm) (OR 10.51; 95% CI: 1.94–56.88) and fluoroscopy use during dilation (OR 22.88; 95% CI: 3.19–164.07) were independent predictors of recurrence within a median follow-up of 22 months.^11^ Our case lacks long-term follow-up, limiting assessment of durability and the need for future interventions.

This case highlights the feasibility of the endoscopic rendezvous approach for long-segment esophageal strictures, particularly in patients at high surgical risk.

## DISCLOSURES

Author contributions: H. Khataniar, IS Sarici, K. Albus, and SE Eriksson contributed to writing, editing, organizing the manuscript, and collecting images. S. Ayazi critically reviewed the manuscript for important intellectual content and provided final approval for publication and is the article guarantor.

Financial disclosure: None to report.

Informed consent was obtained for this case report.
